# Met is involved in TIGAR-regulated metastasis of non-small-cell lung cancer

**DOI:** 10.1186/s12943-018-0839-4

**Published:** 2018-05-12

**Authors:** Mengqin Shen, Xiaoping Zhao, Li Zhao, Liang Shi, Shuxian An, Gang Huang, Jianjun Liu

**Affiliations:** 1 Department of Nuclear Medicine, Ren Ji Hospital, School of Medicine, Shanghai Jiao Tong University, Shanghai, 200127 China; 2 Shanghai Key Laboratory for Molecular Imaging, Collaborative Scientific Research Center, Shanghai University of Medicine & Health Science, Shanghai, 200093 China; 3 Institute of Nuclear Medicine, School of Medicine, Shanghai Jiao Tong University, Shanghai, 200127 China

**Keywords:** TIGAR, Met, Non-small-cell lung cancer, Metastasis, Epithelial-mesenchymal transition

## Abstract

TIGAR is a p53 target gene that is known to protect cells from ROS-induced apoptosis by promoting the pentose phosphate pathway. The role of TIGAR in tumor cell invasion and metastasis remains elusive. Here we found that downregulation of TIGAR reduced the invasion and metastasis of NSCLC cells in vitro and in vivo. Immunohistochemical analysis of 72 NSCLC patients showed that TIGAR and Met protein expression was positively correlated with late stages of lung cancer. Besides, patients with high co-expression of TIGAR and Met presented a significantly worse survival. In addition, we found that Met signaling pathway is involved in TIGAR-induced invasion and metastasis. Our study indicates that TIGAR/Met pathway may be a novel target for NSCLC therapy. It is necessary to evaluate the expression of TIGAR before Met inhibitors are used for NSCLC treatment.

**Electronic supplementary material:**

The online version of this article (10.1186/s12943-018-0839-4) contains supplementary material, which is available to authorized users.

## Background

Cancer statistics collected by the American Cancer Society show that lung and bronchogenic cancer are the leading causes of cancer-related deaths in the United States [1]. Moreover, the trend of lung cancer mortality in China increased markedly and likely to continue to rise [2]. Frequent presence of lung cancer metastases significantly affects efficiency of conventional therapies and induces treatment failure and high mortality [3]. Therefore, there is an urgent need to reveal the underlying mechanism of NSCLC invasion and metastasis.

TP53-induced glycolysis and apoptosis regulator (TIGAR) decreases the level of fructose-2,6-bisphospahte(F-2,6-P2) and subsequently reduces the activity of phosphofructosekinase-1(PFK1). Since PFK1 is the key enzyme in the control of glycolysis, TIGAR leads to glycolysis inhibition and promotes pentose phosphate pathway (PPP) [4]. Tumor metastasis requires metabolic changes to adapt secondary microenvironment [5]. Up-regulation of PPP genes in metastatic lesions compared to primary tumors has been observed in circulating melanoma cells [6], metastatic renal cell carcinoma (RCC) [7] and breast cancer [8]. Therefore, we postulated that TIGAR, as a key regulator of PPP, may be involved in the development of cancer metastasis. There is growing evidence that high TIGAR expression is closely associated with adverse clinical outcomes of patients with multiple types of cancer including chronic lymphocytic leukemia [9], invasive breast cancer [10], stage II and stage III colorectal cancer [11] and nasopharyngeal carcinoma [12, 13]. TIGAR is involved in various biological processes, including metabolism [4], apoptosis, autophagy [14], cell cycle [15], cell death and radiation response. However, the role and mechanism of aberrant TIGAR expression in invasion and metastasis of NSCLC remains unclear.

Met, encoded by MET proto-oncogene, serves as a trans-membrane tyrosine kinase receptor for HGF. The HGF/Met axis mediates a series of biological processes including enhanced proliferation, motility, invasiveness, angiogenesis, morphogenesis, apoptosis and energy metabolism [16]. Over-expressions of HGF and/or its receptor Met have been found in NSCLC cell lines and patients [17–20]. Co-expression of HGF/Met was significantly associated with lymph node invasion [21].

The aim of this study was to explore role of TIGAR in the invasion and metastasis of NSCLC. We analyzed the effect of TIGAR knockdown on motility, invasion, EMT markers and metastasis of NSCLC. In addition, we sought to investigate the relationship between TIGAR and Met in tissues derived from NSCLC patients. Our data indicated that the TIGAR/Met pathway plays an important role in the metastasis of NSCLC and may be a potential target for the treatment of NSCLC.

## Methods

### Cell culture, plasmids, reagents and antibody

All cell lines were purchased from ATCC(Manassas,VA, USA) and maintained at 37 °C in a humidified air atmosphere containing 5%CO2 in Dulbecco’s modified Eagle’s medium supplemented with 10% fetal bovine serum,100 U/ml penicillin and 100 μg/ml Streptomycin(GIBO, Grand Island, NY, USA). PCR-amplified human TIGAR was cloned into pcDNA4TO-Flag/HA. Plasmids were verified by DNA sequencing. Specific Met inhibitor SU11274 (SELLECK), puromycin (Life Technologies), cell cycle rapid detection solution (Dakewe Biotech) was purchased. Anti-Flag M2 (Sigma-Aldrich, St Louis, MO, USA), monoclonal anti-HA (Covance, Deham, MA, USA), anti-TIGAR (Abcam), anti-Met (Cell signaling technology), anti-MMP2 (Abcam), anti-MMP9 (Abcam), Epithelial-Mesenchymal Transition (EMT) Antibody Sampler Kit#9782 (Cell Signaling Technology, Danvers, MA, USA), anti-β-actin (Cell Signaling Technology, Danvers, MA, USA), anti-β-Tubulin (Proteintech) was used according to the Manufacturer’s protocol.

### Immunohistochemistry

All experiments involving human tissues were approved by the Human Assurance Committee of Renji Hospital of Shanghai Jiao Tong University School of Medicine. All procedures involving human specimens were performed with written informed consent according to the Declaration of Helsinki. Only 54 of 72 patients with NSCLC had follow-up records. The follow-up time ranged from 12 to 68 months, with a median time of 38.5 months. For immunohistochemical analyses, sections were de-waxed, hydrated and washed. After microwave antigen retrieval, the slides were treated with 3% H2O2 to block endogenous peroxidase activity and then incubated overnight with anti-TIGAR antibody (1:200) or anti-Met antibody (1:300). The sections were subsequently incubated with the horseradish peroxidase conjugated secondary antibody, followed by treatment with diaminobenzidine chromogen to visualize the signal. The signal intensity of IHC was scored by two experimental researchers without prior knowledge about the patient and sample. The signal intensity was scored on a scale of 0–3 and the percentage of cells with score of 0(0%), 1(1 to 9%), 2(10 to 49%) and 3 (50 to 100%). Immunohistochemistry (IHC) score (0 to 9) was defined as the product of the intensity and percentage of cells. Protein expression was considered positive when IHC score was greater than or equal to 4.

### Construction of TIGAR-shRNA and met-shRNA lentiviral recombinant plasmids, lentivirus packaging and screening stable cells

Cells were infected by lenti-virus with Control shRNA (sense 5’-GATCCTTCTCCG AACGTGTCACGTTCAAGAGACGTGACACGTTCGGAGAATTTTTTG-3′, antisense 5’-AATTCAAAAAATTCTCCGAACGTGTCACGTCTCTTGAACGTGA CACGTTCGGAGAAG-3′), shTIGAR (sense 5’-CCGGGCTTACATGAGAAGTCT GTTTCTCGAGAAACAGACTTCTCATGTAAGCTTTTTG-3′, antisense 5′-AATT CAAAAAGCTTACATGAGAAGTCTGTTTCTCGAGCTCGAGAAACAGACTTC TCATGTAAGC-3′) and shMet (sense 5’-CCGGTCAACTTCTTTGTAGGCAA TACTCGAGTATTGCCTACAAAGAAGTTGATTTTTG-3′, antisense 5’-AATTCAA AAATCAACTTCTTTGTAGGCAATACTCGAGTATTGCCTACAAAGAAGTTGA -3′). Stable TIGAR knockdown and Met knockdown and control cells were screened by puromycin.

### RNA isolation and real-time polymerase chain reaction

Total RNA was isolated using a Trizol kit (Omega, Norcross, GA, USA) and transcribed to cDNA with a cDNA synthesis kit (Takara, Otsu, Japan). Quantitative real-time PCR was performed using SYBR Green PCR Master Mix (Takara) and the transcript levels of genes were detected by using the StepOnePlus Real-Time PCR System (Applied Biosystems, Foster City, CA, USA). Primers used for detection of specific genes are shown in Additional file 1: Table S1.

### Western blot

Cells were lysed into the RIPA buffer containing protease inhibitors by incubating on ice for 30 min followed by centrifugation at 15000 g for 30 min. The extracted proteins were subjected to electrophoresis on 10% SDS-polyacrylamide gels and transferred to PVDF membranes (GE Healthcare, Buckinghamshire, UK), which were blocked and probed with specific primary antibodies with appropriate dilution at 4 °C overnight. The membranes were washed with 1 × TBST three times and were then incubated with the horseradish peroxidase-conjugated secondary antibodies for 1 h at room temperature, followed by three washes with 1 × TBST, the immune-reactive bands were visualized by ECL Plus system (Tanon, Shanghai, China).

### Immunoprecipitation

Extracts of cells overexpressing Flag-tagged TIGAR proteins were incubated with anti-Flag M2 affinity gel for 3 h 4 °C. The samples were analyzed by Western blot after being washed with IP buffer three times.

### Oligonucleotide transfection

SiRNA and negative control that used with transient transfection was designed and synthesized by GenePharma (shanghai, China). Cells were transfected with siRNA using lipofectamine 2000 Reagent (Invitrogen) according to the manufacturer’s protocol. For migration, invasion, RNA extraction and Western blot assays, cells were utilized after transfection 48 h. The sequences of siRNA oligos used in this study are as follows: TIGAR (Sense, 5’-CCUACAGGAUCAUCUAAAUTT-3′; Anti-sense, 5’-AUUUAGAUGAUCCUGUAGGTT-3′), (Sense, 5’-AUGGAGAAACAAGAUUU AA-3′; Anti-sense, 5’-UACCUCUUUGUUCUAAAUU -3′), (Sense, 5’-UGCUGGUA UAUUUCUGA AU-3′; Anti-sense, 5’-ACGACCAUAUAAAGACUUA -3′). Met (Sense, 5’-UCAACUUCUUUGUAGGCAAUA-3′; Anti-sense, 5’-UAUUGCCUACAAAGAAGUUGA-3′), Negative control (Sense, 5’-CCUACGCCACCAAUUUCGU-3′; Anti-sense, 5’-ACGAAAUUGGUG GCGUAGG-3′).

### Wound-healing assays

For cell motility assay, cells were collected and counted using a hem cytometer after trypan blue exclusion. Then the equal amounts of cells were seeded in six-well plates to near confluence. A linear wound was carefully made by a 200ul sterile pipette tip across the confluent cell monolayer, and the cell debris was removed by washing with phosphate-buffered saline and incubated in Dulbecco’s modified Eagle’s medium with 1% fetal bovine serum. The wounded monolayers were then photographed at 0, 24 h after wounding.

### Cell invasion assays

Cell invasion assays were performed in a 24-well plate with 8-μm pore size chamber inserts (Coring, USA). 1x10^5^cells were put into the upper chamber per well with Matrigel-coated membrane. Cells were suspended in 200ul of Dulbecco’s modified Eagle’s medium with 1% fetal bovine serum when they were seeded into the upper chamber. The lower chamber was completed by 600ul of Dulbecco’s modified Eagle’s medium with 10% fetal bovine serum. After incubation for 48 h at 37 °C and 5% CO2, the upper inserts were removed from the plate; the non-invading cells were removed from the membrane. Cells that moved to the bottom surface of the chamber were fixed with 4% formalin for 20 min and stained with 0.1% crystal violet for 30 min. After cleared with PBS, the cells were imaged and counted in at least 5 random fields using microscope.

### In vivo metastasis assays

Five-week-old BALB/C-nu/nu male mice (Shanghai Laboratory Animal Center, Shanghai, China) were used for animal studies. All experimental procedures using animals were in accordance with the guidelines provided by the Animal Ethics Committee of Renji Hospital of Shanghai Jiao Tong University School of Medicine. For the in vivo tumor metastasis assay, 2 × 10^6^ A549 cells stably knockdown TIGAR and negative control were injected into the lateral tail veins of nude mice respectively. After seven weeks, we monitored metastasis foci of nude mouse tumor xenografts by ^18^F-FDG micro-PET/CT. 200-400uCi of ^18^F-FDG were administered intravenously in 200 μL saline, then were scanned by an Super Nova ® micro-PET/CT scanner (Pingseng Healthcare Co, China) and were co-registered with Avatar 1.2 software (Pingseng Healthcare Co, China). Standardized uptake values (SUV) of Regions of interest (ROIs) were assessed after manual definition. After that all nude mice were euthanized and the lungs, livers were harvested at necropsy and fixed in 4% neutral phosphate-buffered formalin. The fixed samples were embed in paraffin and stained with HE (hematoxylin and eosin), Ki67 and TIGAR antibody.

### Flow cytometry analysis

Trypsin digested cells were washed with PBS, suspended with cell cycle rapid detection solution. Cells were analysed by Flow cytometry (Jena, Germany).

### Cell adhesion assay

The 96-well plate was coated with matrigel (40 μg/ml) and incubated 37 °C 1 h. Then using washing buffer (0.1% BSA in DMEM medium) to wash and block plates with blocking buffer (0.5% BSA in DMEM medium). Put 3000 cells in each well. Plate was incubated in 37 °C, 5% CO2 for 2 h. After that to wash plates with washing buffer three times, fix with 4% poly-formaldehyde for 20 min and stain with 0.1% crystal violet for 15 min. Finally cells that adhered matrigel were counted under the microscope three random regions.

### Statistical analysis

All assays were carried out three independent times. All data were statistically analyzed using Graphpad Prism 7 software or Graphpad Prism 6 software (GraphPad Software, San Diego, CA, USA), and presented as mean ± s.e.m., Unpaired two-tailed t-test or ANOVA test was utilized to analyze the difference between the two groups. Spearman’s rank correlation was implemented to determine the correlation between TIGAR and Met proteins in human lung tissue. *P*-values < 0.05 were considered as statistically significant.

## Results

### TIGAR promotes cancer cell motility and invasion in vitro

**Fig. 1:**
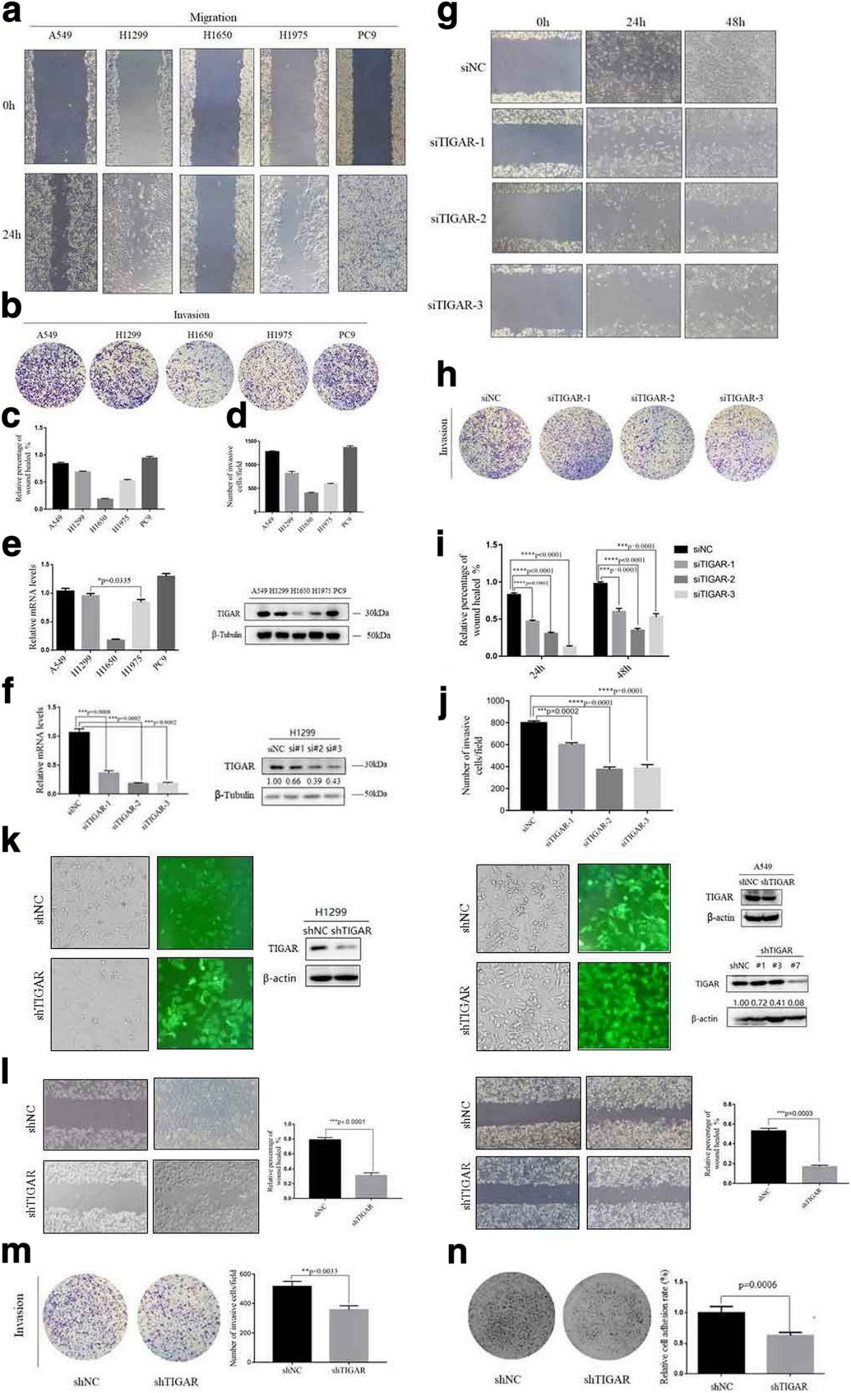
TIGAR expression is increased in high metastatic lung cancer cells, affecting cell migration, invasion and adhesion in vitro. (**a**-**b**) Wound healing assays and Trans-well analysis to determine the migration and invasion of A549, H1299, H1650, H1975 and PC9, respectively. (**c**-**d**) Statistical analysis was to quantify the migratory and invasive ability of five NSCLC cells. (**e**) Real-time PCR and Western blot analysis to determine endogenous TIGAR expression of five NSCLC cells. (**f**) Real-time PCR and Western blot analysis to respectively quantify mRNA and protein expression of TIGAR after transfection with siTIGAR (or siNC as control) for 48h. (**g**-**h**) Cells were transfected with siTIGAR (or siNC as control) for 48h were collected to determine the migration and invasion capability by wound-healing assays and Trans-well assay, respectively. (**i**-**j**) Statistical analysis was to quantify cells migratory and invasive ability upon TIGAR knockdown. (**k**) H1299 and A549 cells were infected with lentivirus with shTIGAR and Green fluorescent protein. To get more effective knockdown, monoclonal cell was to screen in A549. Monoclonal cells A549-shTIGAR#7 was the A549-shTIGAR in following assays. (**l**) Wound-healing assay for H1299 and A549 cells stably knockdown TIGAR or negative control. (**m**) Trans-well invasion assay for A549 cells stably knockdown TIGAR or negative control. (**n**) The effect of TIGAR on cell adhesion activity was assessed in A549

To explore the role of TIGAR in lung cancer invasion and metastasis, we investigated the endogenous levels of TIGAR in five human NSCLC cell lines. These cell lines displayed different metastatic potential (Fig. 1a-1d). Interestingly, TIGAR is markedly expressed in high metastatic cells (A549, H1299, PC9) compared with weakly metastatic cells (H1975, H1650) (Fig. 1e). We then used three pairs of siRNAs to assess the prometastatic effect of TIGAR in NSCLC cell lines (Fig. 1f). TIGAR knockdown significantly reduced cell motility and invasion compared to non-silencing control (Fig. 1g-1j). Subsequently, a lentiviral delivery system was applied to stably knockdown TIGAR in two lines of NSCLC cells (Fig. 1k). We observed consistent results in TIGAR stable knockdown cells (Fig. 1l). Similarly, cell invasion was significantly reduced upon stable knockdown of TIGAR (Fig. 1m). In addition, the effect of TIGAR on the adhesion of the cells to the matrigel was then investigated. We found that in the case of TIGAR knockdown, cell adhesion was reduced by about 35% (Fig. 1n). Taken together, TIGAR promotes cell motility and invasion of NSCLC cells.

### The role of TIGAR in epithelial-mesenchymal transition (EMT)

**Fig. 2:**
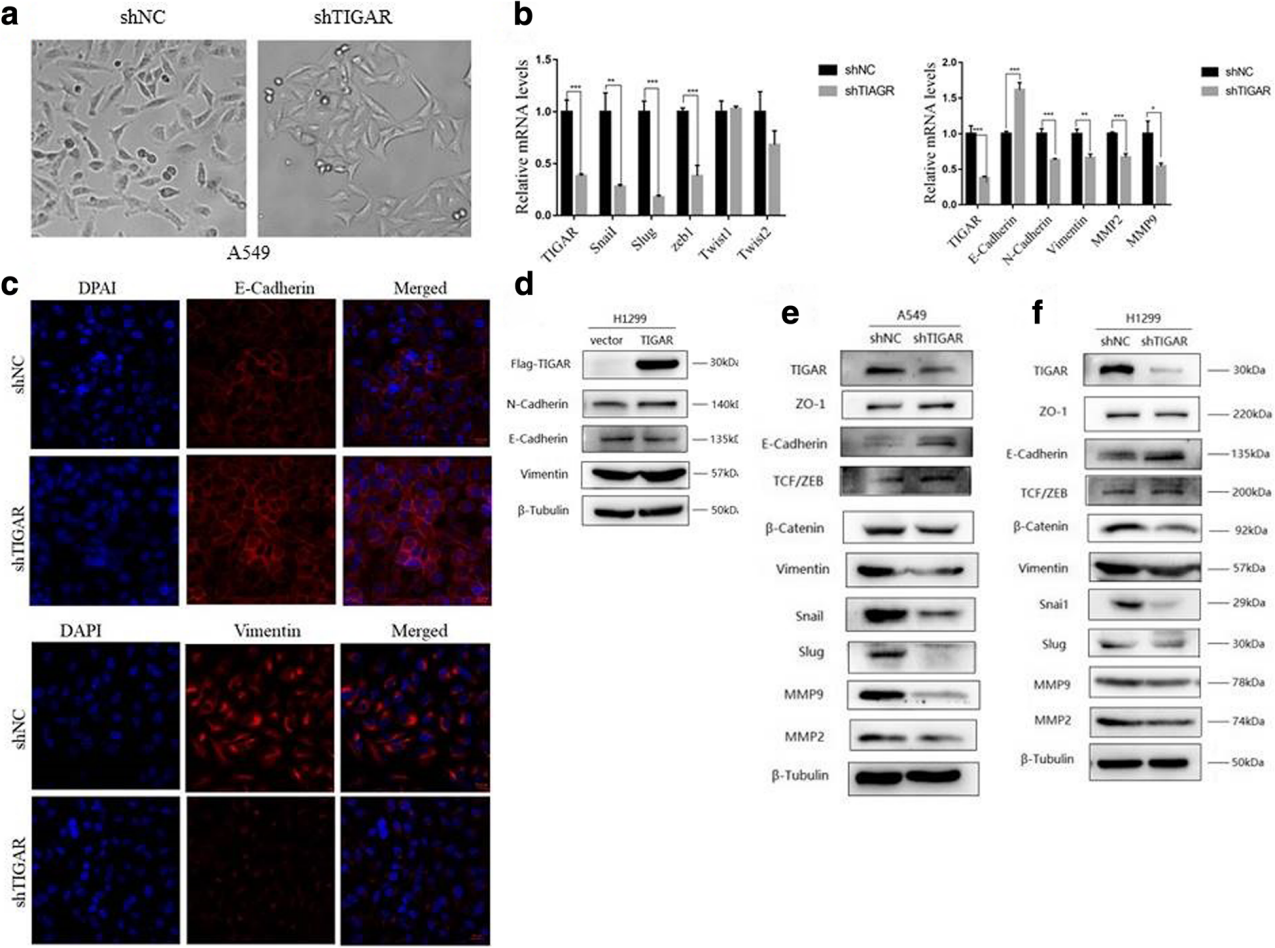
TIGAR affects epithelial-mesenchymal transition (EMT) of lung cancer in vitro. (**a**) Image showed morphological changes of A549 cells stably knockdown TIGAR (x200 magnifications). (**b**) Real-time PCR analysis to qualify the endogenous levels of EMT-associated transcriptional factors (left) and markers (right) after being transfected with siTIGAR (or siNC as control) for 48h. (**c**) The immunofluorescence assay showed that TIGAR interference results in up-regulation of E-Cadherin and down-regulation of Vimentin (x200 magnifications). (**d**) H1299 cells transfected with Flag-TIGAR (or empty vector as control) for 48h. Western blot analysis determined the effect of TIGAR overexpression on mesenchymal marker Vimentin, N-Cadherin and epithelial marker E-Cadherin. β-tubulin was used as an internal control. (**e**-**f**) Western blot analysis to determine the protein levels of EMT markers in two lines of NSCLC cells that stably knockdown TIGAR

We observed a typical morphological change from a narrow spindle to a flattened round, epithelial-like morphology with fewer scattered colonies, while TIGAR was stably knocked down in NSCLC cells (Fig. 2a). Then we evaluated whether TIGAR-induced NSCLC cell migration and invasion was associated with EMT. The mRNA levels of EMT-associated transcriptional factors, SnaiI, Slug and ZEB1 were strongly downregulated in stably knockdown TIGAR cells. The EMT-associated molecule, E-Cadherin mRNA level increased following TIGAR knockdown, whereas mRNA levels of N-Cadherin, Vimentin, MMP2 and MMP9 were decreased (Fig. 2b). Consistently, TIGAR knockdown increased protein levels of E-Cadherin, ZO-1 and decreased protein levels of Vimentin, SnaiI, Slug, β-catenin, MMP2, MMP9 in A549 cell(Fig. 2e). We observed similar phenomena in H1299 cells (Fig. 2f). In addition, TIGAR overexpression increased N-cadherin, Vimentin levels and decreased E-Cadherin level (Fig. 2d). Immunofluorescent staining showed that TIGAR promoted EMT through the decrease of E-Cadherin and the increase of Vimentin (Fig. 2c). The above results indicated that TIGAR promotes NSCLC cell metastasis through EMT regulation.

### TIGAR promotes metastasis of NSCLC cells in vivo

**Fig. 3:**
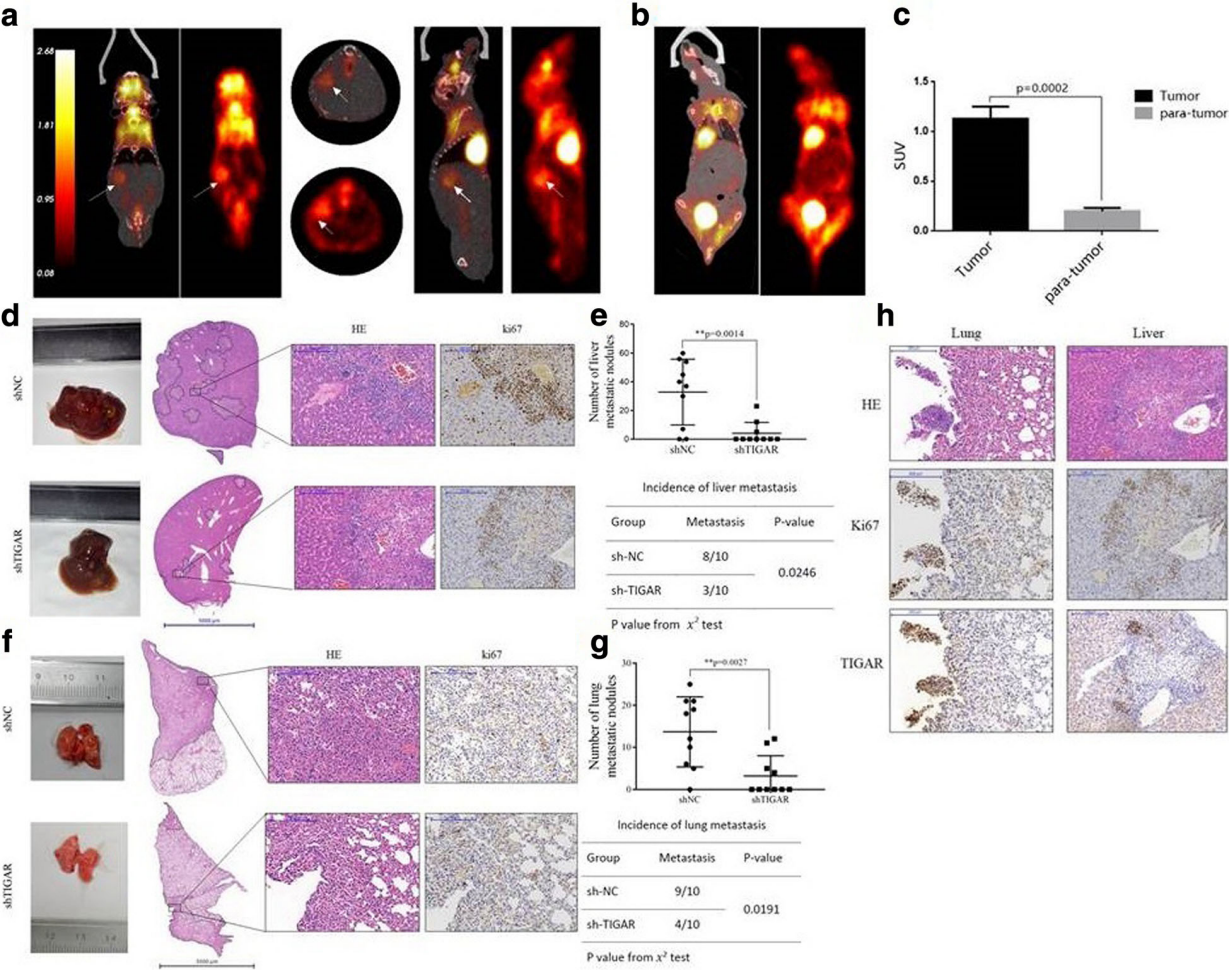
TIGAR promotes metastasis of lung cancer in vivo. (**a**) Representative photos were the coronal, Axial, sagittal co-registered PET/CT and PET images images of a mouse with metastatic lung cancer in the liver from shNC group. Arrows indicate the metastatic tumor on the right of liver, which appears very bright in the images. PET images in color are co-registered with the CT in grayscale. (**b**) The coronal images of a mouse from shTIGAR group didn’t show significant abnormal ^18^F-FDG accumulation. (**c**) Comparison between the tumor and para-tumor in ^18^F-FDG uptake. (**d**-**g**) Tissue sections of intrahepatic (**d**) and pulmonary (**f**) metastases were shown, the quantification of intrahepatic (**e**) and pulmonary (**g**) metastatic nodules was analyzed by Student’s *t*-test, the incidence of intrahepatic (**e**) and pulmonary (**g**) metastasis was analyzed by using the χ2 test in each group (*n* = 10). (**h**) The representative images are hematoxylin and eosin staining, Ki67 staining, TIGAR staining of metastatic foci and precancerous tissue

Then we investigate whether TIGAR also affects lung cancer metastasis in vivo. After 7 weeks of tail vein injection of A549-shTIGAR-cells (shTIGAR group) and A549-shNC cells (shNC group) in nude mice, metastasis was detected by ^18^F-FDG micro-PET/CT imaging. The results showed that shNC group had a strong accumulation of ^18^F-FDG in the liver (Fig. 3a). The maximum standard uptake value (SUVmax) of intrahepatic metastasis was significantly higher than that of paracancerous tissues, suggesting liver metastasis (Fig. 3c). There was no apparent accumulation of ^18^F-FDG in the liver of shTIGAR group (Fig. 3b). Liver and lung histology showed that the number of metastases in shNC group was significantly higher than shTIGAR group (Fig. 3d-3g). In the shNC group, liver metastases occurred in 80% of the mice and lung metastases occurred in 90% of the mice. In the shTIGAR group, 30% of liver metastases and 40% of lung metastases were found (Fig. 3e-3g). Moreover, strong Ki67 expression in the liver of shNC group suggested that TIGAR promoted the growth of metastatic cells (Fig. 3d). Histological analysis of lung tissue showed that shNC group lung metastasis was more apparent than shTIGAR group. However, there was no significant Ki67 expression in lung metastases, suggesting these metastatic cells show indolent growth activity (Fig. 3f). It may take more time to transit from indolent micro-metastasis to overt macro-metastasis. It is worth noting that intrahepatic and pulmonary metastases showed that TIGAR was significantly expressed in the nucleus of cancer cells (Fig. 3h). It is reported that anticancer drugs or hypoxia can cause TIGAR nuclear translocation [22]. Therefore, TIGAR nuclear translocation may represent an adaptation of metastatic cancer cells to environmental changes. Taken together, these results suggest that TIGAR promotes NSCLC metastasis.

### Relationship between TIGAR and met protein expression and clinic characteristics

**Fig. 4:**
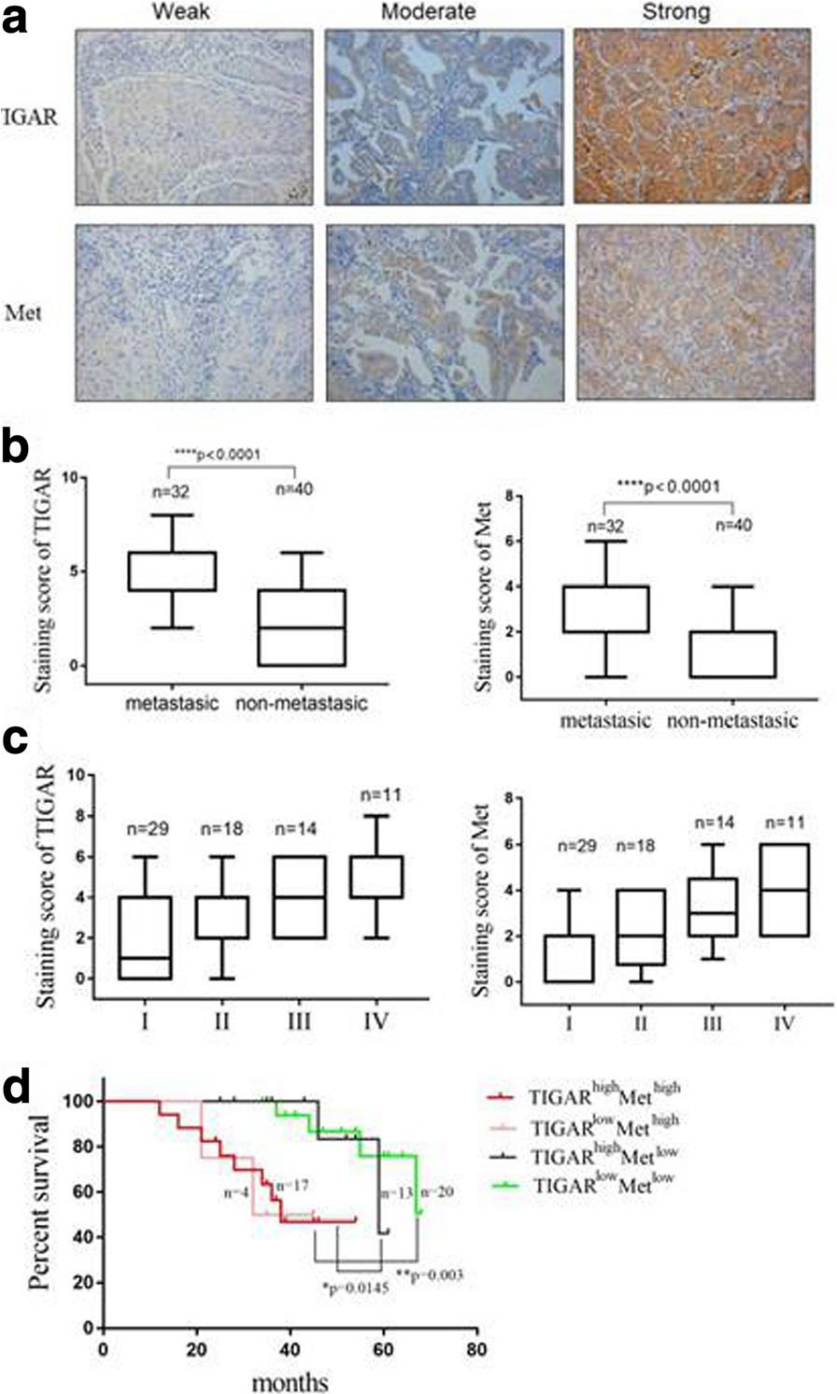
TIGAR is positively correlated with Met, both correlated with advanced clinical stage and tumor metastasis and patient survival. (**a**) Immunohistochemical staining of lung cancer tissue sections showed the different expression of TIGAR and Met. (**b**) Analysis of TIGAR and Met protein expression in metastatic (lymph node metastasis and distant organ metastasis) and non-metastatic tissues from human lung cancer. (**c**) TIGAR and Met expression in different stages of lung cancer tissues. (**d**) Patient survival percentage in TIGAR^high^Met^high^ (Red curve), TIGAR^high^Met^low^ (Pink curve), TIGAR^high^Met^low^ (Black curve) and TIGAR^low^Met^low^ (Green curve) group was showed

IHC analyses were performed on 72 cases of paraffin-embedded lung cancer tissues. Interestingly, we found that TIGAR was associated with Met in NSCLC tissues (*r* = 0.5573, *p* < 0.0001) (Fig. 4a and Additional file 2: Table S2), especially in lymph node and distant organ metastases subgroup (*r* = 0.6576, *P* = 0.0003) (Additional file 3: Table S3). It suggested that the association of TIGAR with Met mainly affected NSCLC metastasis. As shown in Table 1, there was no significant difference in TIGAR and Met expression with age and gender. The level of TIGAR and Met was positively correlated with lymph node metastasis and distant metastasis, suggesting that TIGAR and Met plays an important role in the metastasis of NSCLC (Fig. 4b). High expression of TIGAR was also associated with tumor progression. Additionally, TIGAR and Met expression was higher in stage III and stage IV of NSCLC (Fig. 4c). Compared with patients with low-level TIGAR and Met NSCLC, patients with high levels of TIGAR and Met have poor prognosis (Fig. 4d). Thus, our results suggested that the protein levels of TIGAR and Met is positively correlated in human NSCLC tissues, which predict more malignant characteristics.

**Table 1:** Analysis of correlation between TIGAR or Met protein level and clinic parameters in 72 patients with NSCLC

Protein name		TIGAR					Met		
Characteristics	All cases	Low	High	*P*-value		All cases	Low	High	*P*-value
Participants	72	35	37			72	49	23	
Age				0.7538					0.3726
< 65	46	23	23			46	33	13	
≥65	26	12	14			26	16	10	
Sex				0.5040					0.4921
Female	26	14	12			26	19	7	
Male	46	21	25			46	30	16	
TNM stage				0.0190					0.0012
I	29	20	9			29	27	2	
II	18	8	10			18	11	7	
III	14	5	9			14	7	7	
IV	11	2	9			11	4	7	
Nodes metastasis				0.0005					0.0001
N0	45	29	16			45	38	7	
N1–3	27	6	21			27	11	16	
Distant metastasis				0.0283					0.0143
M0	61	33	28			61	45	16	
M1	11	2	9			11	4	7	
Cancer progression				0.0260					0.0625
No	33	18	15			33	24	9	
Yes	21	5	16			21	10	11	

### TIGAR regulates the expression of met

**Fig. 5:**
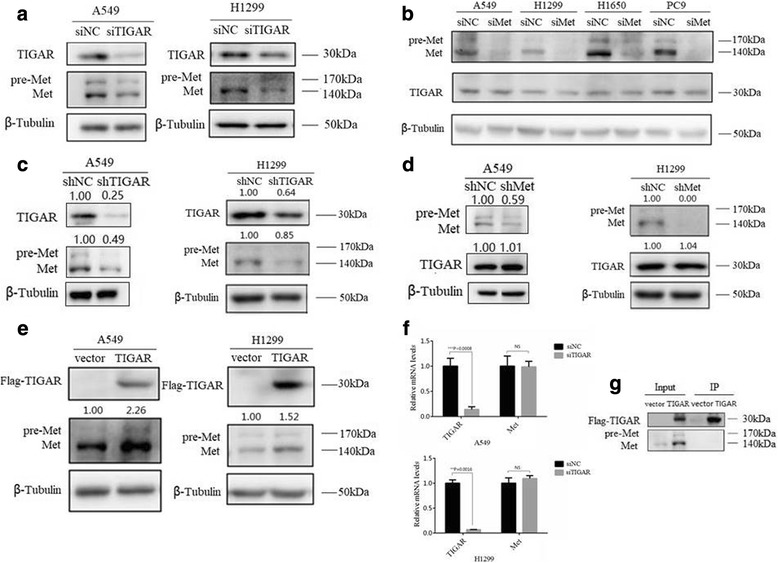
TIGAR regulates the expression of Met protein. (**a**) Analysis of Western Blot showed the effect of TIGAR on Met in A549 and H1299 cells after being transfected with siTIGAR (or siNC as control) for 48h. (**b**) Western Blot analysis showed the effect of Met on TIGAR in NSCLC cells after being transfected with siMet (or siNC as control) for 48h. (**c**) The analysis of Western Blot showed the effect of TIGAR on Met in stably knockdown TIGAR cells. (**d**) The analysis of Western Blot showed the effect of Met on TIGAR in stably knockdown Met cells. (**e**) Overexpression TIGAR enhanced the protein levels of Met in two lines of NSCLC cells after being transfected with Flag-TIGAR (or empty vector as control) for 48h. (**f**) Knockdown TIGAR had not significantly affected on mRNA levels of Met in A549 and H1299 that transfected with siTIGAR (or siNC as control) for 48h. (**g**) Immunoprecipitation (IP) assays showed there was not directly interaction between TIGAR and Met

Met, a trans-membrane tyrosine kinase receptor plays a key role in the invasion and metastasis of cancer. Lui et al. reported that TIGAR is decreased when Met is inhibited in nasopharyngeal carcinoma cells [23]. Then we tested whether TIGAR-regulated invasion and metastasis was correlated with Met. We found that Met was significantly reduced upon TIGAR knockdown (Fig. 5a). In addition, overexpression of TIGAR significantly up-regulated Met levels (Fig. 5e). The regulation of Met expression by TIGAR was also confirmed in TIGAR stable knockdown cells (Fig. 5c). Nevertheless, the results showed instantly (Fig. 5b) or stably (Fig. 5d) knockdown Met had no significant effect on expression of TIGAR. Although the abundance of Met protein can be regulated by a variety of mechanisms, TIGAR regulated Met through a post-transcriptional mechanism because there was no decrease in Met mRNA level upon TIGAR knockdown (Fig. 5f). There was no protein-protein interaction between TIGAR and Met (Fig. 5g), suggesting that an indirect mechanism is occurred underlying the regulation of Met by TIGAR.

### TIGAR promotes cell migration and invasive activity via met

**Fig. 6:**
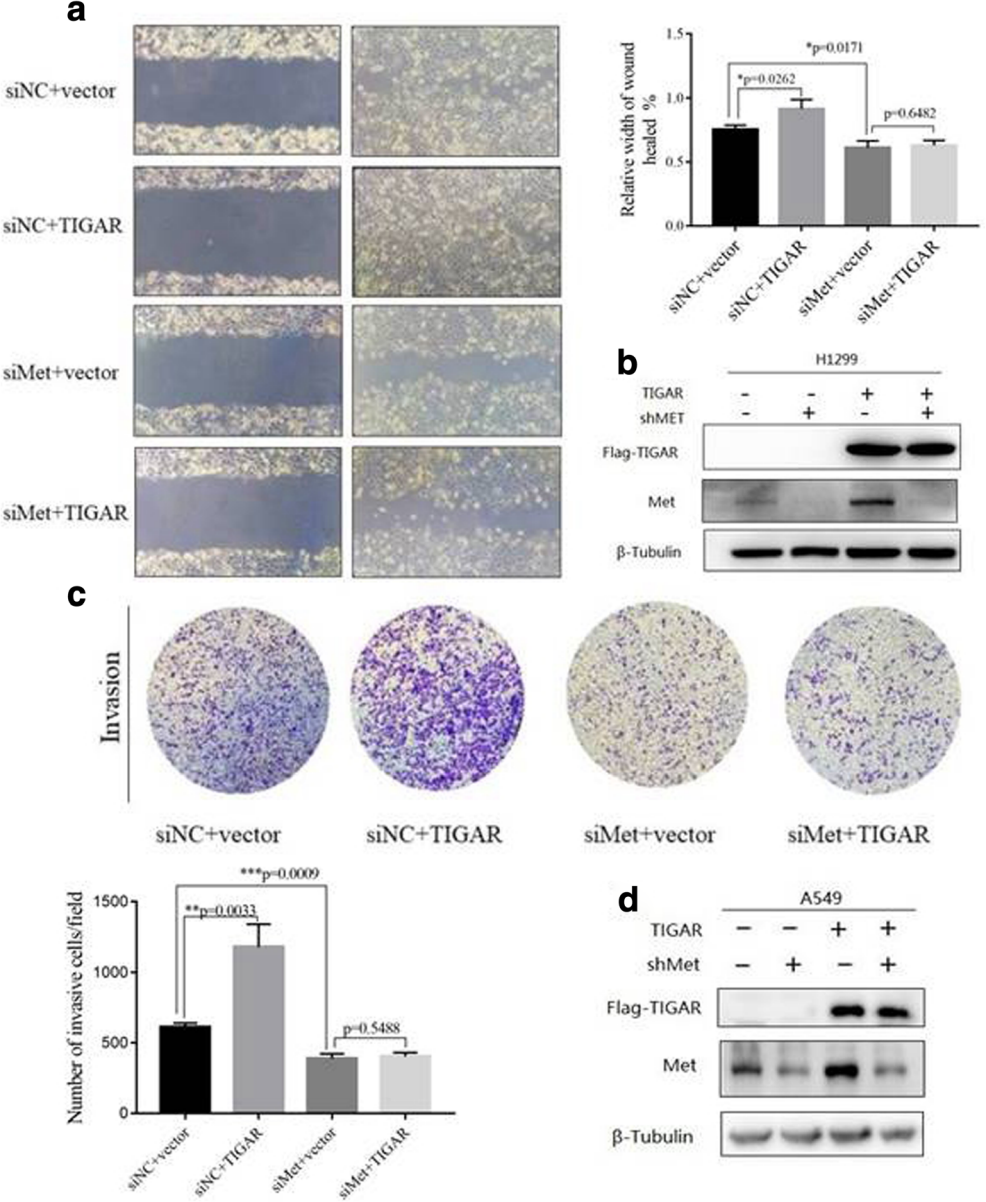
TIGAR promotes migration and invasion of lung cancer via Met. (**a**) H1299 cells were cotransfected with Flag-TIGAR or empty vector (as control) plus siMET or siNC for 48h before wound-healing assay. (**b**) Western Blot showed the protein expression of Met and TIGAR after Met knockdown in TIGAR overexpression in H1299 cells. (**c**) Trans-well assay was performed experiment in A549 cells cotransfected with Flag-TIGAR (or vector as control) and siMet (or siNC as control). (**d**) The protein expression of Met and TIGAR after Met knockdown in TIGAR overexpression in A549 cells was detected by Western Blot

Next, we investigated whether TIGAR promotes cell migration, invasion in a Met-dependent manner. To test this, the effect of TIGAR on metastasis was analyzed in NSCLC cells with or without Met knockdown. Consistent with previous results, TIGAR overexpression strongly increased cell motility, whereas Met knockdown attenuated the effect of TIGAR on cell motility (Figs. 6a-b). Moreover, we found that knockdown of Met abolished the effect of TIGAR in NSCLC cell invasion (Fig. 6c-d). These results indicate that TIGAR promotes cell migration and invasion through Met.

## Discussion

The well-known function of TIGAR is to maintain redox balance through promoting metabolic flux from glycolysis to PPP. There is growing evidence that metastatic lesions may encounter more oxidative stress than primary tumors and therefore require the up-regulation of the PPP pathway [5]. More importantly, a prerequisite for tumor cell metastasis is to increase the metabolic flux of PPP to counteract ROS-induced anoikis upon cell detachment [24]. Therefore, TIGAR may cause cancer metastasis. Here, we found that TIGAR is required for the invasion and metastasis of NSCLC. Mechanistically, Met signaling pathway is involved in TIGAR-induced invasion and metastasis.

TIGAR was initially identified as the transcriptional target of p53, an important tumor suppressor protein [4]. TIGAR is dysregulated in many types of cancer and is involved in in various biological processes such as metabolism, apoptosis, autophagy, cell cycle and cell death [25]. TIGAR promotes intestinal regeneration and tumorigenesis when TIGAR expression is up-regulated and uncoupled from p53 [26]. In addition, TIGAR is highly expressed in patients with chronic lymphocytic leukemia (CLL), regardless of p53 status [9]. Results from p53 null and mutant p53 tumor cell lines indicate that wild-type p53 is dispensable for the expression of TIGAR [26]. Meanwhile, mutant p53 generally exhibits an gain-of-function effect on cancer metabolism [27]. Here we observed that TIGAR contributed to invasion and metastasis regardless of p53 status, since the invasion and metastasis of both p53 wild-type (A549) and p53 null (H1299) cell lines are regulated by TIGAR. In addition, future studies are necessary to elucidate the mechanism by which TIGAR is induced in a p53-independent manner in cancer cells.

TIGAR is involved in various biological processes of cancer cells. The link between TIGAR and invasion of cancer cells has been established by several studies [12, 13]. Indeed, it remains elusive whether TIGAR can promote cancer metastasis in vivo. Here, our in vitro and in vivo data strongly indicate that TIGAR is indispensable for cancer cell invasion and metastasis. TIGAR are regulated through key context-specific or fine-tuning events. A variety of signaling pathways including NF-κB and Met are involved in the role of TIGAR in cancer. Zhao et al. demonstrate that TIGAR-induced nasopharyngeal carcinoma is related to the activation of NF-κB pathway [12]. In contrast, Sinha et al. Found that TIGAR had no effect on NF-κB activation in radiation-mimetic Neocarzinostatin (NCS)-treated cells, but that NF-κB regulates NCS-induced TIGAR expression [28]. The relationship between TIGAR and the NF-κB pathway may depend on the extracellular context and the type of cancer. Our data suggested that Met signaling pathway is involved in TIGAR-regulated invasion and metastasis of NSCLC. Meanwhile, Met inhibition can downregulates TIGAR expression and subsequently decreases NADPH production [23]. Therefore, TIGAR and Met are closely related and mutually regulated in cancer. There is growing evidence that metabolic enzymes are widely involved in signaling pathways. 6-Phosphogluconate dehydrogenase regulates tumor cell migration by regulating receptor tyrosine kinase Met in vitro [29]. Our study may provide evidence that TIGAR may play a similar role in the signaling pathway.

Additionally, we gained insight into the molecular changes of EMT markers, as epithelial to mesenchymal transition (EMT) is a critical step in the invasion-metastasis cascade [30]. We found that TIGAR induced EMT phenotypes in NSCLC cells by activating mesenchymal markers and downregulating epithelial markers. In addition, our results indicate that knockdown of TIGAR reduces the expression of MMP2 and MMP9, which play important roles in the formation of tumor microenvironments and in the promotion of cancer progression and metastasis [31]. However, the mechanism that TIGAR regulated EMT and MMPs needs further exploration.

Metastatic cancer cells undergo significant metabolic rewiring. This metabolic plasticity allows them to survive under restricted conditions [5]. Our study shows that the metabolic enzyme TIGAR promotes the invasion and metastasis of NSCLS. Mechanistically, Met signaling pathway is involved in role of TIGAR in the invasion and metastasis of NSCLC. These findings may suggest that the TIGAR/Met pathway may be a novel target for NSCLC therapy. Met inhibitors are useful for treating NSCLC patients with high TIGAR expression.

## Additional files

Additional file 1: **Table S1.** Primers used in Real-Time PCR. (DOC 41 kb)

Additional file 2: **Table S2.** The expression of TIGAR and Met in lung cancer. (DOCX 15 kb)

Additional file 3: **Table S3.** The expression of TIGAR and Met in metastasis subgroup of lung cancer. (DOCX 15 kb)
