# Supplementary material for: Met is involved in TIGAR-regulated metastasis of non-small-cell lung cancer
Source: Mol Cancer. 2018 May 12;17:88. doi: 10.1186/s12943-018-0839-4 (PMC5948872; doi:10.1186/s12943-018-0839-4)
Supplement: Supplementary file 1 — Table S1. Primers used in Real-Time PCR. (DOC 41 kb) [file 12943_2018_839_MOESM1_ESM.doc]

Additional file 1:Table S1 Primers used in Real-Time PCR

| Genes | Primers |
| --- | --- |
| TIGAR | F: 5'-CTCTGACTGTTGTCCGGCAT-3' |
|  | R: 5'-TGCATGGTCTGCTTTGTCCT-3' |
| MET | F: 5'-AGCAATGGGGAGTGTAAAGAGG-3' |
|  | R: 5'- CCCAGTCTTGTACTCAGCAAC-3' |
| MMP2 | F: 5'-TACAGGATCATTGGCTACACACC-3' |
|  | R: 5'-GGTCACATCGCTCCAGACT-3' |
| MMP9 | F : 5'-TGTACCGCTATGGTTACACTCG-3' |
|  | R: 5'-GGCAGGGACAGTTGCTTCT-3' |
| E-Cadherin | F: 5'-CGAGAGCTACACGTTCACGG-3' |
|  | R: 5'-GGGTGTCGAGGGAAAAATAGG-3' |
| N-Cadherin | F: 5'-TCAGGCGTCTGTAGAGGCTT-3' |
|  | R: 5'-ATGCACATCCTTCGATAAGACTG-3' |
| Vimentin | F: 5'-GACGCCATCAACACCGAGTT-3' |
|  | R: 5'-CTTTGTCGTTGGTTAGCTGGT-3' |
| Snail | F: 5'-TCGGAAGCCTAACTACAGCGA-3' |
|  | R: 5'-AGATGAGCATTGGCAGCGAG-3' |
| Slug | F: 5'-TGTTGCAGTGAGGGCAAGAA-3' |
|  | R: 5'-GACCCTGGTTGCTTCAAGGA-3' |
| ZEB1 | F: 5'-GCCAATAAGCAAACGATTCTG-3' |
|  | R: 5'-TTTGGCTGGATCACTTTCAAG-3' |
| Twist1 | F: 5'-TCCGCGTCCCACTAGCA-3' |
|  | R: 5'-AGTTATCCAGCTCCAGAGTCTCT-3' |
| Twist2 | F: 5'-CGACGAGATGGACAATAAGAT-3' |
|  | R: 5'-CACACGGAGAAGGCGTAGCT-3' |
| Fibronectin | F: 5'-CGGTGGCTGTCAGTCAAAG-3' |
|  | R: 5'-AAACCTCGGCTTCCTCCATAA-3' |
