# Supplementary material for: Met is involved in TIGAR-regulated metastasis of non-small-cell lung cancer
Source: Mol Cancer. 2018 May 12;17:88. doi: 10.1186/s12943-018-0839-4 (PMC5948872; doi:10.1186/s12943-018-0839-4)
Supplement: Supplementary file 2 — Table S2. The expression of TIGAR and Met in lung cancer. (DOCX 15 kb) [file 12943_2018_839_MOESM2_ESM.docx]

Additional file 2: Table S2. The expression of TIGAR and Met in lung cancer

|  | | TIGAR expression | | P-value |
| --- | --- | --- | --- | --- |
|  |  | Weak  n(%) | Strong  n(%) |  |
| Met expression | Weak | 31/35(88.6) | 19/37(51.3) | 0.0006 |
|  | Strong | 4/35(11.4) | 18/37(48.7) |  |
