# Supplementary material for: Met is involved in TIGAR-regulated metastasis of non-small-cell lung cancer
Source: Mol Cancer. 2018 May 12;17:88. doi: 10.1186/s12943-018-0839-4 (PMC5948872; doi:10.1186/s12943-018-0839-4)
Supplement: Supplementary file 3 — Table S3. The expression of TIGAR and Met in metastasis subgroup of lung cancer. (DOCX 15 kb) [file 12943_2018_839_MOESM3_ESM.docx]

Additional file 3: Table S3. The expression of TIGAR and Met in metastasis subgroup of lung cancer

|  | | TIGAR expression | | P-value |
| --- | --- | --- | --- | --- |
|  |  | Weak  n(%) | Strong  n(%) |  |
| Met expression | Weak | 6/7(85.7) | 7/25(14) | 0.0060 |
|  | Strong | 1/7(14.3) | 18/25(72) |  |
